# Provincial Maternal and Child Information System in Inner Mongolia, China: Descriptive Implementation Study

**DOI:** 10.2196/46813

**Published:** 2024-03-25

**Authors:** Yiwei Yan, Congyan Xing, Jian Chen, Yingbin Zheng, Xiaobin Li, Yirong Liu, Zhanxiang Wang, Kai Gong

**Affiliations:** 1 Biomedical Big Data Center The First Affiliated Hospital of Xiamen University Xiamen China; 2 Equipment and Materials Department The First Affiliated Hospital of Xiamen University Xiamen China; 3 Zoe Soft Corp Ltd Xiamen China; 4 School of Health Management Fujian Medical University Fuzhou China

**Keywords:** information system, maternal and child health care, system construction, system implementation, regional health, Inner Mongolia Autonomous Region

## Abstract

**Background:**

After the implementation of 2- and 3-child policies, the rising proportion of high-age and high-risk pregnancies put enormous pressure on maternal and child health (MCH) services for China. This populous nation with an increasing population flow imperatively required the support of large-scale information systems for management. Municipal MCH information systems were commonly applied in developed cities of eastern provinces in China. However, implementation of provincial MCH information systems in relatively low-income areas is lacking. In 2020, the implementation of a regional maternal and child information system (RMCIS) in Inner Mongolia filled this gap.

**Objective:**

This paper aimed to demonstrate the construction process and evaluate the implementation effect of an RMCIS in improving the regional MCH in Inner Mongolia.

**Methods:**

We conducted a descriptive study for the implementation of an RMCIS in Inner Mongolia. Based on the role analysis and information reporting process, the system architecture design had 10 modules, supporting basic health care services, special case management, health support, and administration and supervision. Five-color management was applied for pregnancy risk stratification. We collected data on the construction cost, key characteristics of patients, and use count of the main services from January 1, 2020, to October 31, 2022, in Inner Mongolia. Descriptive analysis was used to demonstrate the implementation effects of the RMCIS.

**Results:**

The construction and implementation of the RMCIS cost CNY 8 million (US $1.1 million), with a duration of 13 months. Between 2020 and 2022, the system recorded 221,772 registered pregnant women, with a 44.75% early pregnancy registry rate and 147,264 newborns, covering 278 hospitals and 225 community health care centers in 12 cities. Five-color management of high-risk pregnancies resulted in 76,975 (45.45%) pregnancies stratified as yellow (general risk), 36,627 (21.63%) as orange (relatively high risk), 156 (0.09%) as red (high risk), and 3888 (2.30%) as purple (infectious disease). A scarred uterus (n=28,159, 36.58%), BMI≥28 (n=14,164, 38.67%), aggressive placenta praevia (n=32, 20.51%), and viral hepatitis (n=1787, 45.96%) were the top factors of high-risk pregnancies (yellow, orange, red, and purple). In addition, 132,079 pregnancies, including 65,018 (49.23%) high-risk pregnancies, were registered in 2022 compared to 32,466 pregnancies, including 21,849 (67.30%) high-risk pregnancies, registered in 2020.

**Conclusions:**

The implementation of an RMCIS in Inner Mongolia achieved the provincial MCH data interconnection for basic services and obtained both social and economic benefits, which could provide valuable experience to medical administration departments, practitioners, and medical informatics constructors worldwide.

## Introduction

In the Sustainable Development Goals issued by the World Health Organization (WHO), the most concerning targets related to good health and well-being were a reduction in the maternal mortality rate (MMR), neonatal mortality rate (NMR), and under-5 mortality rate (UMR), with the purpose of maternal and child health (MCH) improvement [1]. MCH and related measures, such as family planning and immunization, comprise one of the foundation stones for public health [2].

China has emphasized MCH as an essential component of China’s health care system [3]. In October 2016, the Healthy China Action Plan (2019-2030) set clear requirements that the MMR and the NMR should be reduced to 12/100,000 and 5/1000 by 2030 [4]. In the past few years, the MMR of China has dropped from 23.2/100,000 in 2013 to 15.7/100,000 in 2022 [5,6]. After the implementation of the 2-child policy in 2016 [7] and the 3-child policy in 2021 [8], multiparous births exceeded primiparous births nationwide [9]. The rising proportion of high-age pregnancies (maternal age ≥35 years) resulted in a significant increase in pregnancy risks [9], putting enormous pressure on MCH services in China, with over 10 million newborns every year [6].

With the rapid development of medical informatization [10], the information construction for MCH has been a prominent development trend. Thanks to the similar gestation cycle and production inspection process, data types of MCH records are homogenous, which creates a positive precondition for the construction of a maternal and child information system (MCIS). In such cases, localized MCH informatization was launched worldwide. As early as in 1984, the Maternal and Child Health Information Network (MATCH) [11] was created to serve as a pilot project to manage data related to prenatal, child health, family planning, and genetic services in clinics in the state of Ohio in the United States. The South Western Sydney Area Health Service launched the Mother and Infant Network (MINET) in 1997 [12] in order to develop an integrated clinical data network to support a continuum of MCH care, including in-patient, ambulatory, and community-based services. However, all these systems were designed to be used only in a few clinics with limited features and served as a simple tool to record in-diagnosis information, leading to a relatively small application scope.

Given the disparity in economy and medical resources [13], China imperatively requires practical tools to integrate regional medical resources for MCH improvement, especially in low-income areas [14,15]. The increasing population flow with economic migration also demands higher-level resident information sharing in a wider scope [16]. However, although most medical institutions have already set up internal business systems, such as the hospital information system (HIS), the laboratory information system (LIS), and the imaging system, data noninteroperability has become a general problem [17], which poses difficulties for the construction of MCISs and the realization of full coverage of MCH services. In China, the initial attemps for MCISs started at the beginning of 21st century. The first version of the regional maternal and child information system (RMCIS) was implemented in the Xiamen Maternal and Child Health Hospital in 2003. In Shanghai, districts, such as Changning District in 2007 and Songjiang District in 2013, set up their own MCISs. Beijing established a citywide MCIS and a big data platform for MCH information in 2017. Prior interface and data standards for core functions were considered as common solutions to address the issues of data noninteroperability and ensure the realization of basic services. With the increasing requirements for geographical coverage, eastern provinces with high-income economies, such as Guangdong and Guangxi, took the lead in the construction of provincial MCISs. Conversly, due to the relatively poor economic development [18], implementation of provincial MCISs in western provinces is lacking. Challenges are significant because the relatively poor conditions are characterized by a weak economy, inconvenient transportation, a sparse population, and even insufficient medical resources, especially in rural and remote areas [19]. To address the imbalance issue and improve regional MCH services, Inner Mongolia, a western province in China, implemented a provincial RMCIS in 2020.

**Figure 1:**
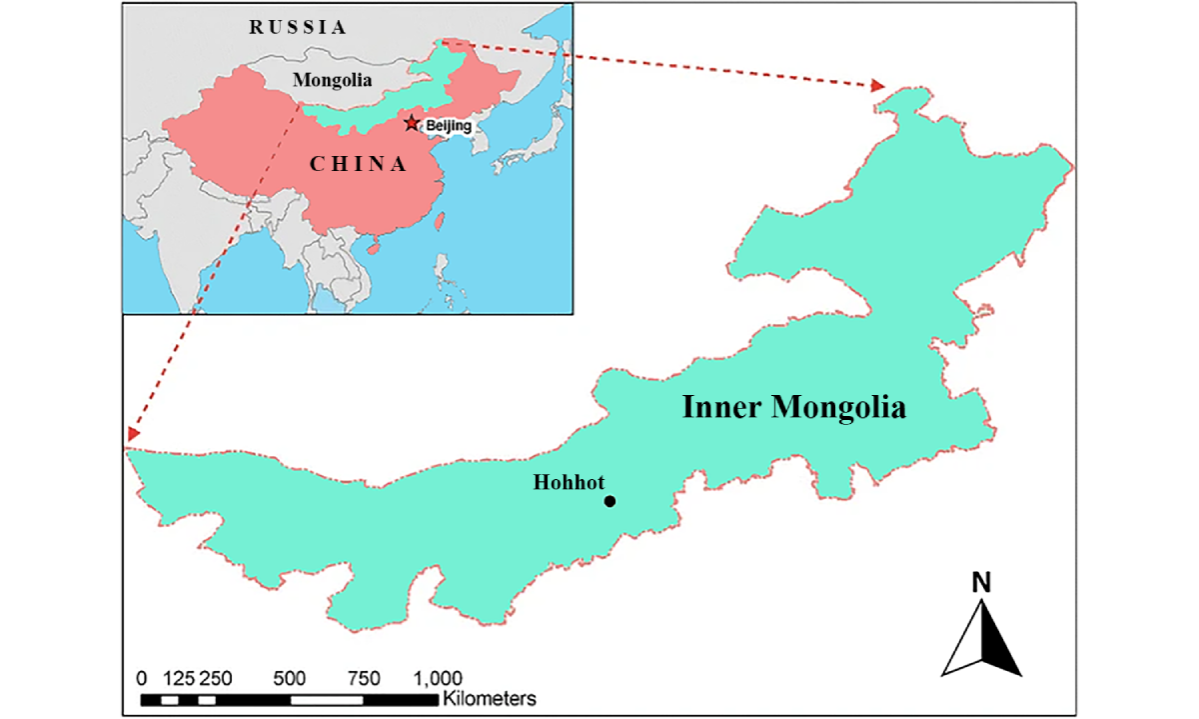
Geographical location map of Inner Mongolia (adapted from Xu et al [21], which is published under Creative Commons Attribution 4.0 International License [24]).

The Inner Mongolia Autonomous Region (hereafter referred to as Inner Mongolia) is an autonomous region of minorities located in north China, with 9 cities and 3 leagues. It covers 1.18 million square kilometers, with 12% of China’s total land area and 24.05 million recorded residents (Figure 1) [20]. Due to the influence of the geographical location, a highly variable climate, and historical and cultural reasons, regional development disparity exists and the average economic development is behind that of eastern coastal regions in the country [21]. In 2021 and 2022, the gross regional product of Inner Mongolia ranked 21st among 34 provinces across the country [22]. This vast territory with a sparse population had a high demand for health service improvement, given the high turnover rate of medical workers in remote places and the limited access to tertiary hospitals for the residents. In such cases, a dense distribution of community health care centers is necessary for universal coverage health, which is the primary aim of the right care [23].

In recent years, Inner Mongolia has vigorously improved its MCH, focusing on the core task of prioritizing children and ensuring the safety of mothers. Before the implementation of the provincial RMCIS in 2020, two allied cities in Inner Mongolia, Wuhai in 2014 and Hohhot in 2017, constructed municipal MCISs for the purpose of MCH service management and data report. MCH outcomes were promoted, with the MMR decreasing from 20.64 to 9.92 for every 100,000 pregnant women and the NMR decreasing from 8.45 to 2.2 for every 1000 pregnant women from 2012 to 2021 [25]. During the 13th 5-year period (2016-2020), the MCH service capacity was also greatly enhanced. The rate of hospitalized deliveries for pregnant women continuously exceeded 99%, the systematic management rate of pregnancies rose from 93.77% to 94.68%, the systematic management rate of children under age 3 years rose from 92.82% to 94.72%, and the rate of screening for women’s diseases rose from 68.84% to 82.73% [26]. Three-level preventive measures against birth defects were implemented, covering urban and rural residents. The maternal prenatal screening rate rose from 49.31% to 88.90%, the newborn disease screening rate rose from 65.04% to 96.86%, and the newborn hearing screening rate rose from 79.96% to 96.23% [26]. The medical service network was extended, maintaining 24,951 medical institutions in total, including 806 (3.23%) hospitals, 1251 (5.01%) community health care centers, 114 (0.46%) MCH hospitals, 1301 (5.21%) MCH and family planning service stations (township health centers), 13,000 (52.1%) village health offices, 15 (0.06%) maternity hospitals, and 2 (0.01%) children’s hospitals [26]. In the 14th 5-year plan, Maternal and Child Health Care Development Plan for Inner Mongolia (2021-2025) [26], with the assistance of the RMCIS, a 5-level MCH service network is expected to cover the entire autonomous region, allied cities, flag counties (cities, districts), soum townships (streets), and gacha villages (communities).

In response to the increasing demand for large-scale service management and data report, the RMCIS would tremendously improve regional MCH with data interconnection. The implementation experience of the RMCIS in Inner Mongolia was a representative case in China, and even worldwide, which filled the gap of provincial MCISs in relatively low-income areas. This paper aimed to describe the construction process and evaluate the implementation effects of the RMCIS in improving provincial MCH in Inner Mongolia, which might provide helpful experience for the construction of RMCISs in other areas.

## Methods

### Role Analysis

The setup of an RMCIS covers extensive MCH services provided in all MCH service institutions. The regional MCH service network usually consists of community health care centers (stations), township health centers, village health clinics (rooms), MCH hospitals, midwifery institutions, child care institutions, and hospitals. In fact, the medical institutions also have the responsibility for other non-MCH–related services, but the design of the RMCIS only focused on MCH services. The collected MCH data are reported to health administration departments. Due to the complex relationships between MCH services and institutions, service role analysis was crucial at the initial stage of establishment.

According to the National Health Commission (NHC) [27], medical institutions can play 7 specific roles in the regional health care system. Due to the particularities of medical institutions’ functional settings, 13 types of institutions were involved in MCH information interworking. The features included in each module for different institutions were clearly set. Furthermore, specific authorizations of different institutions were required for the privacy protection of public health and patients. The role analysis of the MCH service and medical institutions was presupposed in the Technical Program for Construction of Regional Maternal and Child Health Information System (Table 1).

**Table 1:** Role analysis of participating entities and MCH^a^ services.

Institution	Pregnancy registry	Child care	Maternal care	Disease control	Disease management	Medical service	Administrative management
Community health care center	X^b^	X	X	X	X	X	—^c^
Health station	X	X	X	X	X	X	—
Clinic	—	—	—	X	X	X	—
Hospital	—	X	X	X	—	X	—
CDC^d^	—	—	—	X	X	—	—
MCH hospital	—	X	X	X		X	—
Emergency center	—	—	—	—	—	X	—
Blood center	—	—	—	—	—	X	—
Health education institution	—	—	—	—	X	—	—
Health administrative department	—	—	—	—	—	—	X
Health surveillance agency	—	—	—	—	—	—	X
FDA^e^	—	—	—	—	—	—	X

^a^MCH: maternal and child health.

^b^X: available.

^c^Not available.

^d^CDC: Centers for Disease Control and Prevention.

^e^FDA: Food and drug administration.

MCH service roles are classified into basic MCH services and administrative management. Basic health care services include most of the tabulated features and thus relate to the majority of medical institutions. Especially, community health care centers in the communities and health stations in the township take the responsibility of all basic services, which are the cornerstones for the hierarchical diagnosis and treatment system implemented in the country. The RMCIS integrates regional MCH resources and provides intelligent and intuitive tools for health administration departments, medical institutions, and patients. The RMCIS covers medical institutions from community health care centers to township hospitals to municipal hospitals and provides functions including high-risk intelligent assessment, automatic identification of high-risk children, self-collection of outpatient physical signs, and performance appraisal.

### Business Architecture Design

The RMCIS renders continuous services for the entire MCH process. Health care service management is a closed-loop system, which means all actions and records from prediagnosis to in-diagnosis to postevent are traceable, ensuring system-wise efficiency, security, and quality.

A standard pregnancy care process protocol was set up by the NHC, according to the Pregnancy and Childbirth Health Care Work Administrative Regulation and the Pregnancy and Childbirth Health Care Work Principles [28] of 2011. To protect pregnant women’s rights stipulated in the Maternal and Child Health Law of the People's Republic of China [29], the whole process of pregnancy and childbirth care was divided into 4 consecutive steps: preconception care, antenatal care, intrapartum care, and postnatal follow-up.

Preconception care mainly refers to a series of health services for a couple preparing for pregnancy, including health education and consultation, preconception medical examinations, health status assessment, and guidance.

Antenatal care is launched between the conception confirmation and the delivery, referring to health education and consultation, antenatal medical examinations, general examinations, obstetric examinations, and ancillary examinations (ie, basic examinations and recommended examinations). The regulation requires that every pregnant woman receive at least 5 antenatal visits: 1 first visit and at least 4 revisits. If conception is confirmed using ultrasonography, the pregnant woman is registered and managed in the system and receives a copy of the *Maternal and Child Health Handbook* (MCHH) developed by the Japan International Cooperation Agency originally in 1948 [30].

The frequencies of antenatal visits differ in every trimester. During the first trimester (ie, before the 13th gestational week) and the second trimester (ie, from the 13th to the 27th gestational week), pregnant women only receive antenatal care every 4 weeks, while in the third trimester (ie, after the 28th gestational week), the frequency reduces to 1 or 2 weeks. Each pregnant woman undergoes necessary basic and optionally recommended examinations, depending on her specific pregnancy conditions during antenatal care.

Five-color management for pregnancy risks was proposed by the Department of Maternal and Child Health of the NHC [31]. Pregnant women are classified into 5 levels of pregnancy risk using colors: green (low risk), yellow (general risk), orange (relatively high risk), red (high risk), and purple (infectious disease), as shown in Table 2. Pregnant women without risks are recommended to get registered and obtain a copy of the MCHH for recording in community health care centers in the first trimester, which is considered an early filing rate and is used during the performance appraisal of those centers. After the 21st gestational week of pregnancy, women who have never completed any maternity checkups in community health care centers should get registered in hospitals to undergo a proper medical examination. However, if a woman’s pregnancy risk is high (ie, yellow, orange, red, or purple), arrangements will be made in a tertiary hospital even before the 21st gestational week. Regarding delivery, pregnant women are suggested to make an appointment for a bed in the first trimester (before the 14th gestational week) in a community health care center, but only hospitals are qualified to help them give birth.

**Table 2:** Risk levels and types in 5-color pregnancy risk management.

Risk color	Risk level	Risk types
Green	Low risk	No pregnancy complications
Yellow	General risk	Age≥35 or ≤18 years, genital malformation, pelvic stenosis, etc
Orange	Relatively high risk	Age≥40 years, severe preeclampsia, severe anemia, etc
Red	High risk	Dangerous placenta previa, placental abruption, severe anemia, etc
Purple	Infectious disease	Viral hepatitis, syphilis, AIDS, etc

Intrapartum care refers to a process of comprehensive and dynamic assessment of maternal health, monitoring of mother and fetus, and prevention and treatment of complications during labor and delivery.

After delivery, the postnatal follow-up includes health care during hospitalization, postnatal visits for the mother and the newborn, and postnatal checks. Primary care workers offer 2 visits, one within 3-7 days and the other within 28 days after delivery. The postnatal check for mothers should be performed in a qualified maternity institution 42 days after delivery.

Following the whole preconception care, childhood follow-ups for newborns are conducted in the 1st, 3rd, 6th, 8th, 12th, 18th, 24th, and 36th months during the first 3 years. Later, follow-ups are conducted annually by kindergartens until the child becomes 6 years old.

**Figure 2:**
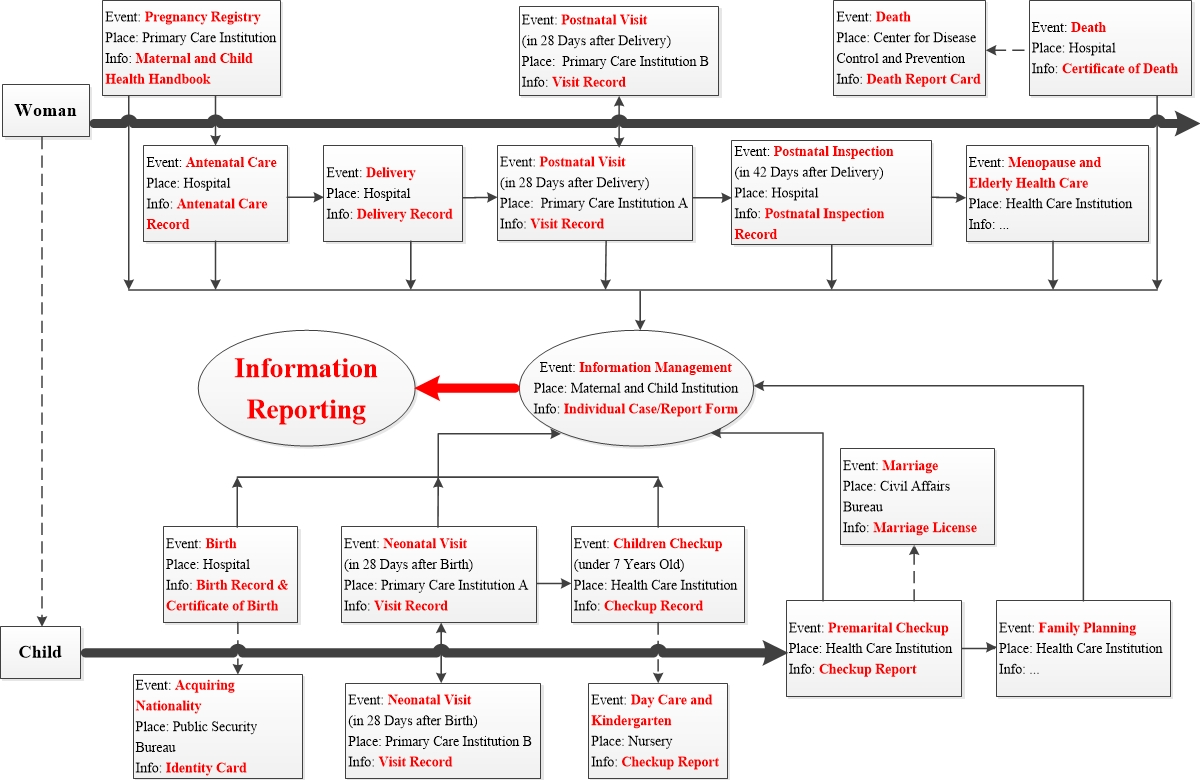
Business architecture design for the information reporting process.

The information reporting process of the RMCIS was designed according to the aforementioned closed-loop process. In addition to pregnancy and childbirth care, other maternal care services, such as premarital checkup, family planning, cervical cancer and breast cancer (CC and BC) screening, and childhood follow-up, are also included in this system. Every event occurs in the way of individual information forms created in the system. Based on the role analysis, each event takes place in a different medical institution, but all the data are uploaded to the same information system. In addition, the RMCIS is linked to other health data systems, such as public security bureaus, ensuring that vital statistics data, such as birth and death certificates, are accurately collected (Figure 2).

### Functional Modules and Services

The essence of an RMCIS is to achieve data interconnection between medical institutions and provide coherent services for the whole health care process based on standardization. Core services were classified into 4 dimensions: basic health care services, special case management, health support, and administration and supervision. Compared to the basic health care services, which are the foundation for medical institutions and cover the whole life cycle of patients, other intelligent supplementary features of pregnancy and childbirth care, such as pregnancy risk surveillance and special case management of high-risk children, guarantee that patients with different medical needs receive proper treatments. Policy making and clinical decision support help medical workers comprehend the health status of patients and accordingly provide prescriptions. Databases for special cases, such as maternal anemia, weight gain during pregnancy, and high-risk newborns, are also built based on this system.

The RMCIS provides health support to users with self-service, but it still has great potential for development. The broadest application of self-service is pregnancy registry prefiling, which reduces the repetitive work of medical workers and ensures the authenticity of patients’ personal information. Patients can fill their personal information in the prefilling forms through mobile apps or official WeChat accounts and conveniently complete pregnancy registry later in community health care centers. Common features (eg, health encyclopedia, daily service information and policies, automatic reminders, medical records, and examination result queries) and extensive services (eg, nutrition assessment, psychological assessment) have also been added to the mobile apps. In addition, daily personalized pregnancy knowledge and appointment reminders can be set in official WeChat accounts. Thanks to the telemedicine services provided by online hospitals, patients can further initiate interactive communications with health care experts online. Finally, the latest technologies, such as the internet of things (IoT), allow remote fetal heart monitoring to be applied broadly, even during an epidemic.

As mentioned in the *Role Analysis* section, most administration and supervision functions are directed to administrative departments, such as the NHC. The administrative departments can then extract data and display MCH surveillance indicators (maternal mortality and child mortality under age 5 years and neonatal birth defect surveillance) through queries. In this way, comprehensive statistics based on MCH can be easily gathered, contributing to period report writing. However, for administrative departments in medical institutions, special case management and supervision are crucial for the treatment of high-risk patients, while performance appraisal is a tool to assess medical workers.

**Figure 3:**
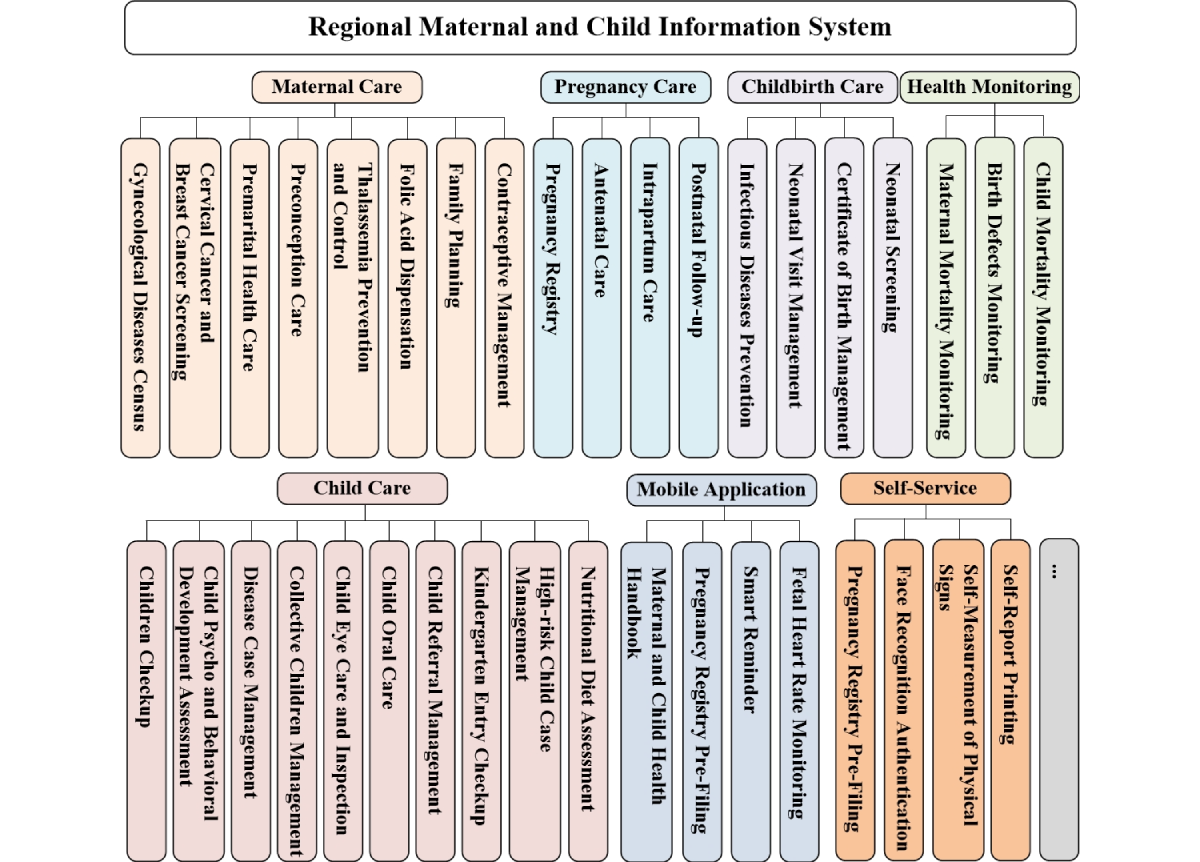
Functional module diagram for the regional maternal and child information system (RMCIS).

Based on 4 core dimensions (basic health care services, special case management, health support, and administration and supervision), 10 modules were set up in the system: (1) maternal care, (2) pregnancy care, (3) childbirth care, and (4) child care for basic health care services; (5) mobile app and (6) self-service for health support; (7) health monitoring, (8) system management, (9) data center, and (10) other for the health administration department and the maintenance team. The maternal care module covers most of the services except pregnancy and childbirth care (which is divided into antenatal care and neonatal care), while the child care module only includes childhood follow-up services (Figure 3).

### Intelligent Health Management and Decision Support

As concrete health care services can be divided into specific modules, intelligent health management and decision support are embedded in every feature of the RMCIS to assist medical workers with reasonable prescriptions. These intelligent services using new techniques make the RMCIS distinct from a simple information system.

The RMCIS can identify and assess the risk of a pregnant women based on her physical signs, report results, and *International Classification of Diseases, Tenth Revision* [32] diagnosis. Subsequently, the system estimates the pregnancy risk level and automatically implements 5-color management. Normal or low-risk pregnant women are further distinguished from high-risk patients by gestational diabetes, hypothyroidism, iron deficiency anemia, twin pregnancies, preeclampsia, etc, so that the system can make an intelligent diagnosis more precisely. Since the management of each patient is a closed-loop system, doctors can easily look up previous maternity checkup results according to the timelines and make a diagnosis with the help of the RMCIS. For high-risk pregnant women, the RMCIS reminds doctors and patients to carry out special case management and track on a regular basis.

The RMCIS also assesses children’s growth status according to the measured data and alerts whether a child has a high-risk growth problem (wasting, obesity, anemia, etc). The system is interfaced with other in-hospital systems to extract data of physical signs and laboratory results, automatically calculates the child’s growth and development status, and draws a WHO standard growth monitoring chart to assess the development level. In this manner, doctors can intuitively observe the child’s growth status and give reasonable advice with intelligent health management guidance from the RMCIS. Special cases are highlighted in the system according to WHO and 9 Chinese provincial and municipal standards, effectively reducing deaths caused by untimely detection or resuscitation of high-risk diseases.

Furthermore, the traditional outpatient physical sign collection equipment has been transformed to an automatic one with the IoT technique. For instance, the automatic electronic blood pressure meter can be highly interconnected with intelligent equipment and an HIS, which can automatically upload blood pressure data to the system. It can also automatically fill in the patient’s physical signs form, without manual entry, and thus simplifies the maternity checkup process. In addition, the remote fetal heart rate monitoring services have been extended to communities and families, closing the gap in out-of-hospital monitoring. Pregnant women can carry out fetal heart monitoring at home according to doctors’ advice and upload the real-time monitoring data through their mobile phones. Hence, nursery stations can immediately uncover emergency conditions.

### Technical Design

In the system design, feasibility, flexibility, robustness and scalability, and maintenance were considered as the basic design principles of system integration [33]. With reference to the guidelines of the NHC [34,35], the RMCIS was designed and constructed to achieve the whole life cycle management of MCH services for residents.

The RMCIS was deployed at a specialized network, while mobile services were deployed on the internet. Logical isolation separated the internet from the specialized network through firewalls and bastions. The system has realized docking with HISs, citizens’ health platforms, management systems of primary health care institutions, and other systems, standardizing the management of MCH services in covered medical institutions and realizing the sharing and exchange of MCH resources. Large distributed databases, such as ORACLE (recommended for expandability and compatibility) and SQLServer, were supported, and centralized storage was adopted, with the builder providing the database server and specifying the storage location. In November 2020, the concept of the cloud-based maternal and child service was proposed and promoted by the NHC [16], and the latest technique (cloud computing) was recommended to be applied in MCH services. Data could be stored in either the cloud or the local data center, which improved the energy efficiency ratio of resource usage. Faster cross-institutional and cross-regional business collaboration and data interoperability became available. Three-layer browser/server (B/S) system architecture was adopted in the software based on service-oriented architecture consisting of an application layer, a service layer, and a data layer. Each layer only focused on its own tasks to successfully separate business from logic and client from database, which feasibly met system design requirements. The advantage of the B/S system architecture was obvious because users had the ability to access the system directly using the web browser, providing easy maintenance and high flexibility.

### Budget Planning

The construction cost of the RMCIS was based on the provincial or municipal population number, since the population number determines the number of medical institutions and the workload of the system going live and docking. In addition, different function modules led to discrepant prices. The cost for medium-to-large cities with a population of more than 5 million was about CNY 4-6 million (US $555,609-$833,414), while that for small cities with a population of less than 5 million was about CNY 3-4 million (US $416,707-$555,609). This cost included system development, implementation, etc. The maintenance cost is 10% of the total cost per year.

### Sustainability

To ensure the sustainability of the RMCIS, several actions were taken. Operation and maintenance management systems were established for all aspects, from funds to personnel. Data collection channels and data management regulations require fixed personnel and procedures to ensure stable and safe data sources. The data backup management system implements regular data backup and ensures that the system can be immediately accessed and restored after failure. The maintenance system monitors operation behavior to ensure correct operation status and avoid attacks, and system emergency plans can maintain business continuity in the case of an emergency. The maintenance fund guarantee system ensures that a certain amount of maintenance investment is kept to promote system updating and upgrading.

An operations and maintenance team was formed and provided by the technology company to ensure operation of the system without disruption. System operation and maintenance mainly include business operation consultancy, data maintenance, software maintenance, and hardware platform maintenance. Remote scheduled manual checking at regular intervals is the basic safeguard. If any system failure occurs, a text message and an email are directly sent to the duty personnel for notification. The time limit for a response to failure is 1 hour, and the repair time limit is 1 hour for major failures and 4 hours for general failures. Technical support is even offered to arrive on-site within a time limit to solve any problem.

As the lead department of the regional MCH system, the regional health care commission took the responsibility for user training. Centralized training and counseling for users was organized after system implementation. In addition, documents and videos of operation tutorial, as well as online support, were provided for users in need.

### Data FLow

The RMCIS implemented in Inner Mongolia provides 2 modes of data collection, depending on the management requirements of local health commissions: direct upload mode and front-end processor collection mode. In the direct upload mode, medical institutions directly upload data through the web service interface after strict encryption. In the front-end processor collection mode, the business system of each medical institution first pushes data to the local front-end server, and then the collection client module automatically collects the data and submits them to the provincial maternal and child data center. The RMCIS supports the front-end processor collection mode with automatic data collection and submission. This way, connecting with the HIS, the LIS, the imaging system, financial systems, and other systems, the RMCIS deployed in individual medical institutions is able to collect data from health record information, tests, images, and reports of related MCH services. In addition, the RMCIS obtains information from personalized mobile apps and official WeChat accounts.

Following guidelines of the NHC [32,36], the data collection process of the RMCIS strictly follows standardized criteria to avoid ambiguous information exchange, including data elements, basic data sets, and data dictionaries. In real MCH business activity, relevant information and data are recorded in the form of record forms (eg, birth certificate, newborn disease screening record form, basic maternity registration form), making up 1 or more basic data sets (eg, birth certificate data set, newborn disease screening data set, maternal health services and high-risk management data set). The collection of standardized smallest units of health record data in unified formats, that is, data elements (eg, name, gender code, date of birth, detailed address), constitutes a data set as well as a record form. Taking the record form of birth certificates as an example, the corresponding data set is the birth certificate data set, which consists of the data elements newborn name, newborn gender code, newborn birth date and time, birthplace, etc.

To ensure the quality of collected data, the system automatically monitors and calibrates the uploaded data based on the data quality monitoring rules: accuracy, completeness, consistency, repeatability, and timeliness. In the face of various construction levels of HISs and the quality of data across the province, the provincial MCH data center needs to formulate a unified data set, data dictionary, business forms, quality control rules, etc.

All data are classified by category and regional distribution and displayed vertically from top to bottom, which means higher authorities are able to access the data of the corresponding affiliates. The RMCIS defines permissions for each user of each organization according to their role and authorizes management to access, download, and view statistical reports. In addition, case access and collaborative sharing functions are also provided, that is, as long as the resident’s health card is inserted when they visit a health care institution within the province, the health care workers are able to access the resident’s MCH information and records online with prior consent.

For data display and visualization, a surveillance module was developed to display relevant indicators, statistical reports, and graphs. Specific reports and graphs form the *Inner Mongolia Annual Report on Maternal and Child Health* [37] based on reporting specifications, including the annual report on maternal health care and health status, the annual report on the health care and health status of children under 7 years, the annual report on the health status of nonfamily children and pregnant women, the annual report on premarital health care, the annual report on the health care work of nurseries and kindergartens, the annual report on the work of midwifery service organizations, the annual report on the monitoring of deaths of children under 5 years, and the quarterly report on the number of perinatal babies. The RMCIS also provides the functions of single or multiple conditional combination queries, as well as printing and exporting query results. These MCH data visualization functions can help with health management decision-making and assist policy makers in determining future regulation outlines for further promotion.

### Ethical Considerations

The First Affiliated Hospital of Xiamen University Ethics Committee approved this study (approval number SL-2021KY044-01). All the users consented to sharing the information for using the developed RMCIS. Privacy protection and data security were emphasized with technical support from technicians even before the system was implemented. Specific security solutions were determined by Technical Program for Construction of Regional Maternal and Child Health Information System.

A 3D information security protection system was built to ensure confidentiality, integrity, availability, and controllability spanning the hardware, software, network, system, data, application, and management. The security protection level was set to level 3 because any potential vulnerabilities might influence the social order and public confidence. Since the RMCIS was deployed on the government affairs’ cloud, which met the requirements of information security level 3, the physical environment security, computing environment security, security boundary protection, communication network protection, and security management center required for the system all met the requirements.

To ensure data security, security access functions, including permission management, identity authentication, and access control management, were applied. Permission management allows the RMCIS to be controlled no matter what operations a specific user had undertaken, including editing, adding, and deleting. Identity authentication is also one of the main prevention and protection strategies to prevent the core information system from illegal access. Furthermore, access control management guarantee that permissions in the system are determined by user roles. Only specific users, such as government staff, are allowed to access functions with higher permission levels, while the rest of the users can only access modules of their own sections. For safety requirements, the system provides log management, recording modification and log queries. In terms of network security, the system supports links, such as private networks or virtual private networks, to ensure secure data transmission. Encryption and decryption technology, identity authentication, and security policy were used to guarantee the security of data transmission. Lastly, data set disaster recovery, customer data platform disaster recovery, data backup, and graded multiclass disaster recovery were designed and implemented in this study.

### Data Collection and Statistical Analysis

This study collected summary data of the RMCIS implementation in 12 cities of Inner Mongolia. Since the system was implemented in Inner Mongolia since January 1, 2020, the speed of informatization construction of the 12 cities varied, so earlier data were not completely uploaded into the system. Partial data from Wuhai, Ordos, and Alaxa were absent because of inaccessibility. The experimental data used in this study only included those collected from Wuhai, Hohhot, and Tongliao in 2020, with partial 2021 data of Wuhai and Alaxa missing. Until October 31, 2022, all 12 cities in Inner Mongolia completed data interoperability with the RMCIS. Thus, the limitations of the data included data missing for extended services in some cities with a short duration.

The study followed the descriptive implementation methodology designed to demonstrate the construction process and implementation effects with the sample size of functional modules and the usage count of services. For distribution of specific users, the basic characteristics of pregnant women (ie, maternal age, gestational week at registration, maternal height, maternal weight, pre-pregnancy BMI, gestational week at delivery, gravidity, education level, parity, current smoking, current alcohol consumption, mode of delivery, multiple gestations) and that of newborns (ie, birth length and birth weight) were collected. Five-color management results were presented in a pie chart for ratios and a histogram for the top 10 factors as an example of high-risk management. The usage count per year for services (ie, pregnancy registry, delivery, newborn, premarital checkup, and CC and BC screening) and pregnancies (ie, total pregnancies and high-risk pregnancies) was displayed to demonstrate its application effects in each city. All these data were securely stored by Zoe Software Engineering Co, Ltd (Xiamen City), which cooperates with medical institutions to run the RMCIS. The company deidentified these data and constructed an internet environment for secure analysis, obtaining data according to the principle of minimum availability. After analyzing the data in the intranet security environment, the analysts exported the analytical results rather than the underlying data.

Project summary data, including duration, labor cost, and cost for each period, were provided by the project team of Zoe Software Engineering Co, Ltd. Other basic information, such as the MMR, NMR, and UMR, were shown on the website of the People's Government of Inner Mongolia Autonomous Region.

Descriptive analysis was performed using Microsoft Excel 2016. In addition, the aggregation and analysis of the top 10 factors of five-color management were performed using Python 3.10.7.

## Results

### Cost Summary

Project planning was initiated at the end of 2018, and the RMCIS was implemented in January 2020, with a duration of 13 months. The labor cost was 210 person-months, and the cost was CNY 8 million (US $1.1 million). In the 4-year maintenance, the first year was free and the later 3 years cost CNY 800,000 (US $111,142; 10% of the implementation cost) per year, leading to a total cost of CNY 10.4 million (US $1.4 million). See Table 3 for details.

**Table 3:** Duration, labor cost, and system construction cost.

Project period	Duration (months)	Labor cost (person-months)	Cost (million CNY)
Planning	2	10	0.5 (US $69,463)
Design, and research and development	8	140	5.5 (US $764,111)
Deployment	3	60	2.0 (US $277,858)
Total	13	210	8.0 (US $1.1 million)

### Data Characteristics

In Inner Mongolia, the RMCIS covered 278 hospitals and 225 community health care centers in 12 cities. The main modules launched included pregnancy care, childbirth care, and maternal care. A total of 221,772 pregnancy registries, with a 44.75% (n=99,241) early pregnancy registry rate, were recorded in the system, along with 1,417,066 records of antenatal visits and 145,863 pregnancies for intrapartum care and delivery care. In addition, records of 147,264 newborns were maintained in the childbirth care module, with medical information including weight, height, newborn disease screening, etc. From January 1, 2020, to October 31, 2022, 56,430 people underwent premarital checkups and 256,659 women underwent CC and BC screening (Table 4).

**Table 4:** Usage count of main modules and services in the RMCIS^a^ (January 1, 2020-October 31, 2022).

Module and services^b^			Data	Value
**Pregnancy care**				
	Pregnancy registry	Pregnancies, N		221,772
	Pregnancy registry	Early pregnancies, n/N (%)		99,241/221,772 (44.75)
	Antenatal visit	Records, N		1,417,066
	Antenatal visit	Pregnancies, n/N (%)		202,879/1,417,066 (14.32)
	Intrapartum care	Pregnancies, n/N (%)		147,510/202,879 (72.71)
	Intrapartum care	Deliveries, n/N (%)		145,863/147,510 (98.88)
	Intrapartum care	High-risk pregnancies, n/N (%)		83,376/147,510 (56.52)
Childbirth care			Newborns, N	147,264
**Maternal care**				
	Premarital health care	People, N		56,430
	CC and BC^c^ screening	Women, N		256,659

^a^RMCIS: regional maternal and child information system.

^b^For each service, the duration was from January 1, 2020 to October 31, 2022.

^c^CC and BC: cervical cancer and breast cancer.

The pregnancy registry, antenatal visits, and intrapartum care services had been put to use, while antenatal screening, postnatal follow-up, and kindergarten checkup data were still deficient. Note that the data of CC and BC screening were maintained in the RMCIS only from January 2022, because this service was applied using other systems previously.

The median maternal age was 30 (lower quartile [Q_L_]-upper quartile [Q_U_] 27-33) years, the median gestational week at pregnancy registration was 14 (11-22) weeks, and the median gestational week at delivery was 39 (38-40) weeks. The median birth length and birth weight of newborns were 50 (50-51) cm and 3300 (Q_L_-Q_U_ 2990-3600) g (Table 5). Gravidity refers to the number of pregnancies, including current and past ones. A total of 20,165 (19.52%) pregnancies were multiparous, which meant a woman had more than 1 delivery of a fetus at or after the 24th gestational week, while the rest of the pregnancies (n=83,134, 80.48%) were nulliparous (no birth history). Only 926 (0.44%) pregnant women had a habit of smoking, and 724 (0.35%) currently consumed alcohol. A total of 48,135 (43.35%) deliveries were conducted by caesarean section, although WHO set a warning cutoff for the rate of cesarean section as less than 15% [38]. See Table 6 for the details.

**Table 5:** Numerical characteristics of pregnant women and newborns recorded in the RMCIS^a^ (January 1, 2020-October 31, 2022).

Numerical characteristic	Median (Q_L_-Q_U_)^b^	Total pregnant women/newborns, n	Missing values, n (%)
Maternal age (years)	30 (27-33)	246,167	0^c^
Gestational weeks at registration	14 (11-22)	244,743	1424 (0.58)^c^
Maternal height (cm)	162 (158-165)	246,146	21 (0.01)^c^
Maternal weight (kg)	62 (55-70)	242,128	4039 (1.64)^c^
Pre-pregnancy BMI (kg/m^2^)	23.83 (21.33-26.84)	242,128	4.39 (1.64)^c^
Gestational weeks at delivery	39 (38-40)	147,510	0^d^
Gravidity (times)	1 (1-3)	147,510	0^d^
Birth length (cm)	50 (50-51)	147,171	0^e^
Birth weight (g)	3300 (2990-3600)	147,171	0^e^

^a^RMCIS: regional maternal and child information system.

^b^Q_L_: lower quartile; Q_U_: upper quartile.

^c^N=246,167 (number of pregnancies in antenatal care).

^d^N=147,510 (number of pregnancies in delivery care).

^e^N=147,171 (number of newborns in childbirth care).

**Table 6:** Categorical characteristics of pregnant women recorded in the RMCIS^a^ (January 1, 2020–October 31, 2022).

Categorical characteristics			Pregnant woman, n (%)		Missing values, n (%)
**Education level**			120,665 (100.00)		125,502 (50.98)^b^
	No education or semiliterate	352 (0.29)		—^c^	
	Primary education	4777 (3.96)		—	
	Junior secondary education	29,253 (24.24)		—	
	Secondary education	16,312 (13.52)		—	
	Undergraduate	63,173 (52.36)		—	
	Postgraduate	6795 (5.63)		—	
**Parity**			103,299 (100.00)		44,211 (29.97)^d^
	Nulliparous	83,134 (80.48)		—	
	Multiparous	20,165 (19.52)		—	
**Current smoking**			208,916 (100.00)		37,251 (15.13)^b^
	Yes	926 (0.44)		—	
	No	207,990 (99.56)		—	
**Current alcohol consumption**			208,916 (100.00)		37,251 (15.13)^b^
	Yes	724 (0.35)		—	
	No	208,192 (99.65)		—	
**Mode of delivery**			111,042 (100.00)		36,468 (24.72)^d^
	Vaginal delivery	62,907 (56.65)		—	
	Caesarean section	48,135 (43.35)		—	
**Gestations**			147,510 (100.00)		0^b^
	Singleton	147,053 (99.69)		—	
	Multiple	457 (0.31)		—	

^a^RMCIS: regional maternal and child information system.

^b^N=246,167 (number of pregnancies in antenatal care).

^c^Not applicable.

^d^N=147,510 (number of pregnancies in delivery care).

### Pregnancy Risks and Factors

**Figure 4:**
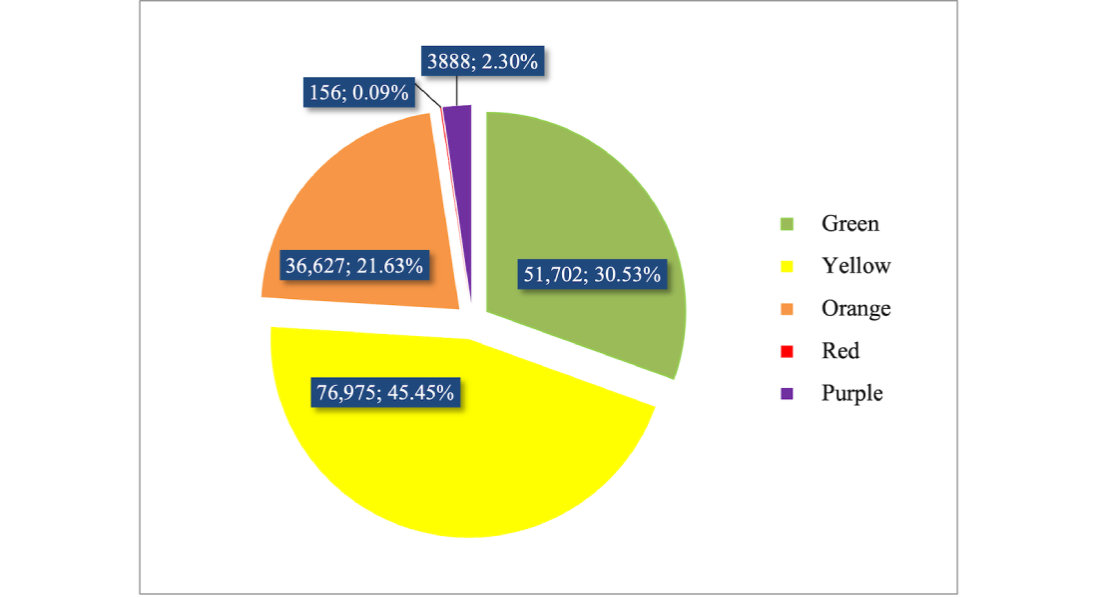
Numbers and percentages in 5-color pregnancy risk management (January 1, 2020–October 31, 2022).

The RMCIS provided statistics for 5-color management. In total, 51,702 (30.53%) pregnancies were marked green (no pregnancy complications). Pregnancies marked yellow (general risk) accounted for the largest proportion (n=76,975, 45.45%), and 36,627 (21.63%) pregnancies were marked orange (relatively high risk). Only 156 (0.09%) pregnant women were marked red (high risk). Furthermore, pregnancies marked purple (infectious disease) were 3888 (2.30%) in total (Figure 4).

**Figure 5:**
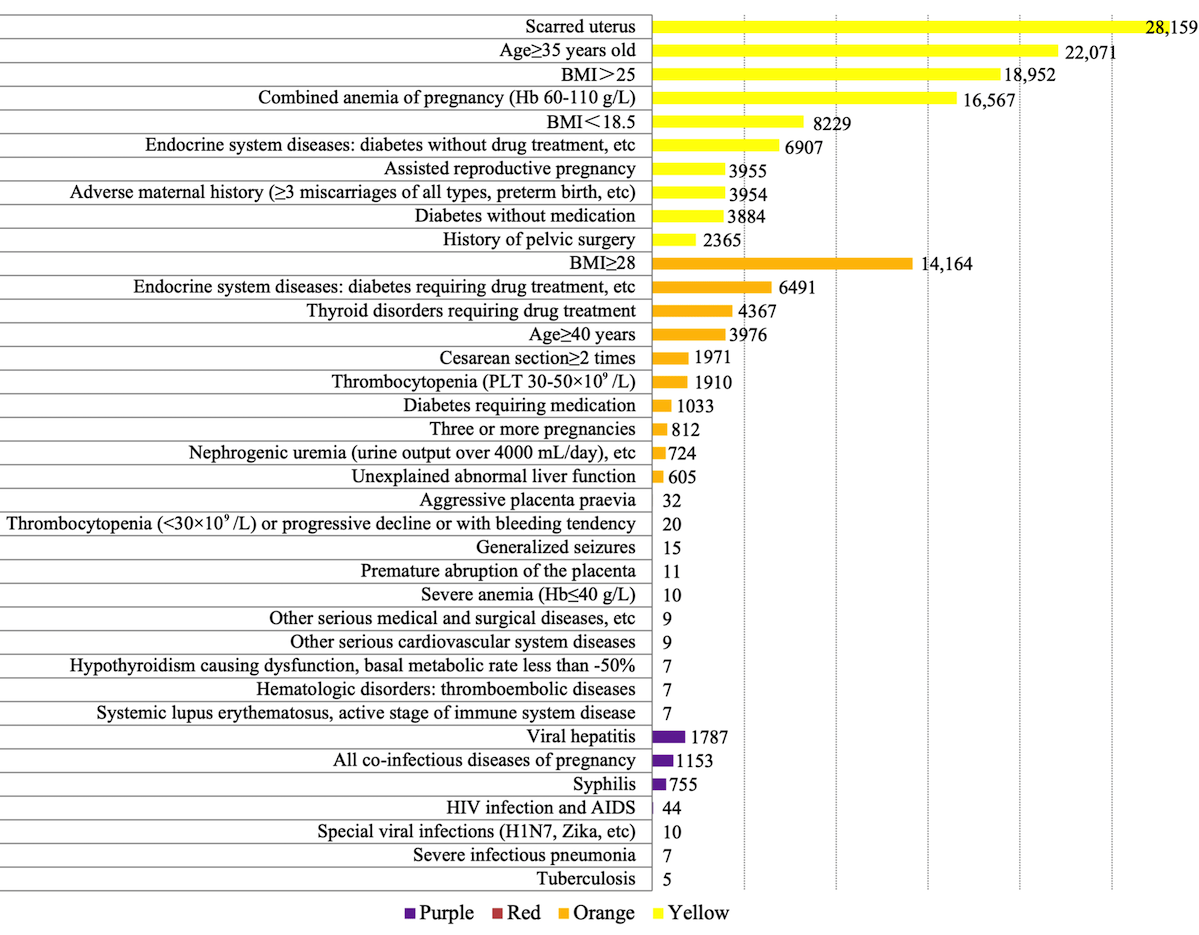
Top 10 factors and number of high-risk pregnancies (yellow, orange, red, purple) from January 1, 2020, to October 31, 2022..

For yellow risk, the top 4 factors accounting for 74.54% of cases were scarred uterus (n=28,159, 36.58%), age≥35 years (n=22,071, 28.67%), BMI>25 (n=18,952, 24.62%), and combined anemia of pregnancy (hemoglobin 60-110 g/L; n=16,567, 21.52%). For orange risk, BMI≥28 (n=14,164, 38.67%) accounted for almost 40% of cases and the 3 reasons were endocrine system disease (eg, diabetes requiring drug treatment, thyroid disease, pituitary prolactinoma, etc; n=6491, 17.72%), thyroid disorders requiring drug treatment (n=4367, 11.92%), and age≥40 years (n=3976, 10.86%). Red risk only accounted for 156 (0.09%) cases, and aggressive placenta praevia (n=32, 20.51%) was the most influencing factor. For purple risk (infectious disease), viral hepatitis (n=1787, 45.96%), all coinfectious diseases of pregnancy (n=1153, 29.66%), and syphilis (n=755, 19.42%) were the top 3 factors, accounting for over 98% of cases (Figure 5).

### Implementation and Popularity

Major modules and services were put into use in 2020, while the minor ones were not due to system transformation and various other reasons.

The 3 main modules put into use were pregnancy care, childbirth care, and maternal care. For pregnancy registry and delivery, the number of pregnant women doubled from 2020 to 2022. The number of newborns increased by almost 1000 every year. Premarital checkup data started to be recorded from 2021, and CC and BC screening was recorded from 2022, since previous data were maintained using other systems alternatively (Table 7).

In 2020, only 3 cities chose to apply and upload MCH data to the RMCIS, while in 2021, Wuhai and Alxa still used their own municipal MCH systems, and parts of cities only uploaded partial data to the system. Yet, now, all 12 cities have participated and contributed to the RMCIS construction in Inner Mongolia. Until October 2022, 132,079 pregnancies were registered in the system, with 65,018 (49.23%) high-risk pregnancies, compared to only 32,466 pregnancy registrations in 2020. The number of pregnancies managed in the system increased by 157.9% in 2021 and 57.74% in 2022 (until October). The popularity of RMCIS implementation is high, and the result of the whole regional MCH data interconnection is significant (Table 8).

**Table 7:** Annual usage count of major services (January 1, 2020-October 31, 2022).

Year	Pregnancy registries, n (%)	Deliveries, n (%)	Newborns, n (%)	Premarital checkup, n (%)	CC and BC^a^ screening, n (%)
2020	45,374 (46.25)	30,211 (30.79)	22,520 (22.95)	0	0
2021	87,245 (47.20)	46,942 (25.40)	23,609 (12.77)	27,044 (14.63)	0
2022^b^	91,106 (19.36)	68,358 (14.52)	25,231 (5.36)	29,350 (6.24)	256,659 (54.53)

^a^CC and BC: cervical cancer and breast cancer.

^b^Until October 31, 2022.

**Table 8:** Annual and high-risk pregnancies in 12 cities of Inner Mongolia (January 1, 2020–October 31, 2022).

City	2020		2021		2022a	
	Pregnancies (n=32,466), n (%)	High-risk pregnancies (n=21,849, 67.30%), n (%)	Pregnancies (n=83,734), n (%)	High-risk pregnancies (n=39,466, 47.13%), n (%)	Pregnancies (n=132,079), n (%)	High-risk pregnancies (n=65,018, 49.23%), n (%)
Ordos	0	0	116 (0.19)	31 (0.08)	43,962 (33.28)	19,756 (30.39)
Baotou	0	0	4124 (4.93)	1977 (5.01)	5336 (4.04)	2866 (4.41)
Hohhot	27,806 (85.65)	20,301 (92.92)	24,330 (29.06)	16,697 (42.31)	20,834 (15.77)	10,678 (16.42)
Chifeng	0	0	36,596 (43.71)	11,756 (29.79)	17,753 (13.44)	9684 (14.89)
Tongliao	325 (1.00)	75 (0.34)	11,980 (14.31)	6323 (16.02)	15,063 (11.40)	8089 (12.44)
Hulunbuir	0	0	2413 (2.88)	833 (2.11)	6312 (4.78)	2455 (3.78)
Xilin Gol	0	0	847 (1.01)	450 (1.14)	3981 (3.01)	1916 (2.95)
Bayan Nur	0	0	112 (0.13)	59 (0.15)	5976 (4.52)	3531 (5.43)
Ulanqab	0	0	203 (0.24)	54 (0.14)	1180 (0.89)	231 (0.36)
Wuhai	4335 (13.35)	1473 (6.74)	0	0	2995 (2.27)	1368 (2.10)
Xingan	0	0	3013 (3.60)	1286 (3.26)	7982 (6.04)	4114 (6.33)
Alxa	0	0	0	0	705 (0.53)	330 (0.51)

## Discussion

### Principal Findings

This study described the construction process (system architecture, functional modules, and construction costs) of the RMCIS in Inner Mongolia and demonstrated the implementation effects with popularity (the usage of main services and 5-color management of pregnancy risks) and challenges. Based on the data collected, the RMCIS implementation was found to have many social and economic benefits, providing a feasible solution to RMCIS construction.

### Social Benefits

The first social benefit was the achievement of MCH medical resource integration, providing supervision tools for governments, data sharing for medical institutions, and easy access to medical resources for residents. Basic services of pregnancy care, childbirth care, and maternal care were put into use. The whole MCH process was optimized through data interconnection and fast resource allocation (eg, hospital transfer, delivery bed appointment) [17]. Clinical decision support and intelligent health management helped health care providers make decisions and improve patient care [39]. The RMCIS also provided clear insight for users through calculated indicators, tables, and charts, leading to advanced performance appraisal tools for governments and administration departments. The quality, efficiency, and equity of regional MCH services were improved for the purpose of universal health coverage [40].

Furthermore, the RMCIS was linked to other public health data sets (eg, birth and death certificate data sets). Studies on MCH can be conducted based on the data collected by the system, and a few studies with a specific historical background have become available. Recently, the research priorities in MCH shifted to COVID-19 during the pandemic era worldwide [41]. Regarding domestic policies in China, the effects of the 1-child policy on MCH were also a hot topic [42]. Indicators or MCH outcomes can be compared within different regions of China or with other countries. Otherwise, since the system tries to cover the whole MCH care process for patients, long-term follow-up studies on sequela after delivery (eg, pelvic floor dysfunction after vaginal delivery [43] or uterus rupture after cesarean delivery [44]) or rehabilitation therapy effects on children with birth defects [45] are feasible. Thanks to large-scale data, techniques, such as big data, provide the basic foundation to build models to predict maternal outcomes. Some factors leading to death or birth defects can be explored [46,47]. Moreover, rare cases can be investigated due to the construction of special case databases. As Figure 2 shows, services, such as thalassemia prevention and control, neonatal defect screening, high-risk pregnancy case management, and high-risk children case management, were included in the RMCIS, and thus special case databases were built. After high-risk patients are detected during diagnosis and treatment, they are included in special case management and attract specific attention. Based on special case data, potential scientific studies focusing on rare cases can be conducted. Maternal anemia [48], gestational diabetes with weight gain [49], and high-risk newborn outcomes [50] are hot topics studied by medical workers. In addition, information about birth defects, children with medical handicaps, and growth retardation on stature or brain power is recorded and linked to other systems. Based on this, a new MCH data source profile can be built. For instance, a new data source profile, the Xiamen Registry of Pregnant Women and Offspring (REPRESENT), was developed [51], linking the 4 major health care data platforms: Resident Healthcare Management Platform, Primary Healthcare Management Platform, Electronic Healthcare Records Platform, and Maternal and Child Health Management Platform. The profile provided further potential uses of pregnancy registry data.

### Economic Benefits

The most important economic gain was the reduction in the operating costs for health care institutions and the medical fee for patients [17]. The workflow was further optimized within various institutions at all levels, and staff was streamlined. The problems of inner-hospital duplicate examinations and medications caused by the inability to share information were mitigated, directly reducing the wastage of health resources. Combined with the reform of medical insurance currently promoted in China [52], the medical fee charged became reasonable and acceptable. Finally, the operation efficiency of entire health care services was enhanced so that the sunk costs for institutions and patients decreased.

### Challenges and Strategies

First, the biggest barrier to start-up was the huge financial requirement, since an MCIS does not generate direct economic effects but otherwise requires high operation and maintenance costs. The technology company seeks economic benefits, while medical institutions and governments only accept cost-effective ones. This benefit leverage between these parties can potentially hinder the migration of the system. Especially in low-income areas, deficient expenditure can lead to insufficient technical equipment, a lack of training opportunities, a high turnover rate of medical workers, and demotivation of patients. With a relative shortage of medical workers who even have to wear multiple hats, the use of helpful information systems can free them from tedious and repetitive tasks so that they can engage in more rewarding work. Investment at once with a low maintenance cost in exchange for a high degree of informatization helps with rationalization of staffing, MCH service quality improvement for residents, and more straightforward management for governments.

Second, a key factor affecting the RMCIS construction was technology compatibility and data interoperability, similar to the issues in other areas. Making the system compatible with other internal systems and public health databases demands high coordination between institutions and technology companies. On a policy level, strong policy support of governments is indispensable for participating parties to collaborate with clear objectives. On a technical level, standardized criteria of data collection should be predesigned. In fact, the implementation of the system does not indicate full coverage of MCH services. Prior interface standards for core functions are required to ensure the realization of basic services. Other interfaces for extended services (eg, postnatal follow-up, kindergarten checkups) can be further expanded and improved according to the experience in Inner Mongolia.

Third, the demotivation of health care workers was common during the implementation process. Since some medical institutions kept paper records, the application of the RMCIS was considered inferior than that of other basic information systems by staff. In addition, some of the health care workers preferred to provide medical instructions by experience, without intelligent decision-making support. The conversion of treatment habits was challenging. In Inner Mongolia, in addition to helpful tutorials and routine training opportunities, administrative tools, such as performance appraisal, were also used to regulate health care workers’ behavior.

Fourth, after operation, requirements of rapid upgradation or optimization for the system were frequently raised to meet new needs, demanding capacity and efficiency from the maintenance team. During the CONVID-19 pandemic, measures, such as epidemiological investigation, real-time reporting of fever clinics, and polymerase chain reaction tests, were required to be added. The technical team was skilled, with ample experience. The RMCIS was used in 2 provinces (Inner Mongolian Autonomous Region and Jiangxi Province), in 6 cities (Xiamen, Fujian; Longyan, Fujian; Pingxiang, Jiangxi; Xinyu, Jiangxi; Jingdezhen, Jiangxi; and Dongying, Shandong) and 7 MCH hospitals. Engineers of the technology company and local technical teams are participating in development and maintenance work. The operation and maintenance management system (as mentioned in the *Sustainability* section) of the RMCIS is referenceable for other areas.

Fifth, network security and privacy security are nonnegligible. The network security law requires information security level protection and level assessment for compliance [53]. It also requires the implementation of sensitive data classification and classification protection. Protecting the data from attacks is a task. Considering the balance of convenience and security, B/S architecture with a private network was deployed in the RMCIS. Each medical institution can use the private network instead of the internet to access the system. This technical solution will provide a higher level of network and privacy security.

Finally, the direct upload mode of data collection (as mentioned in the *Data Flow* section) can lead to some issues. The RMCIS cannot automatically collect and submit data, requiring a data entry team to manually upload MCH records to the system. Every time after a routine production inspection, pregnant women need to take extra efforts to look for a data entry team member to enter her records in the MCHH, which demotivates patients. Since some information can be urgent, the manual uploading process impedes the data flow. Staff even have to call patients for verification if any data are missing. Such inconveniences occurred due to the management requirements of some local health commissions, resulting in duplicate work. Thus, the front-end processor collection mode used in Inner Mongolia is highly recommended for migration.

Due to the homogeneity of the maternal and child service process, the migration of the RMCIS seems feasible. In conclusion, governments should provide strong policy and financial support; medical infomatic constructors need to ensure data interoperability, security, and sustainable maintenance; administration departments in medical institutions should provide routine training opportunities and performance appraisal to motivate doctors; and medical workers are encouraged to keep pace with new techniques. These countermeasures have proven to be effective for the implementation of the RMCIS in Inner Mongolia, which might inspire other areas and even other countries.

### Limitations of the Study

The study has several limitations. The period for which data were collected was relatively short, covering less than 3 years. Cities except Wuhai and Hohhot had not used a municipal MCIS before the implementation of the RMCIS, leading to missing data in 2020 and 2021. Extended services (eg, antenatal screening, postnatal follow-up, and kindergarten checkups) are still unused in the system, as further data interoperability with interfaces is awaited. Due to concerns of data security, the data collected in this study were deidentified and privacy-related data, including infectious diseases, were omitted. The artificial techniques used in the RMCIS are still immature, and the accuracy of clinical decision remains to be verified. Further studies can include benefits and outcomes, such as the impact on MCH outcomes, efficiency of health care delivery, patients’ system management rate, and patient satisfaction, to evaluate the implementation effects of the RMCIS.

### Conclusion

Based on the field study in Inner Mongolia, regional MCH improved through the effective implementation of an RMCIS. The system showed significant social and economic benefits, providing supervision tools for governments, provincial MCH data sharing for medical institutions, clinical decision support for health care workers, real-world data for researchers, and easy access to medical resources for residents. We can foresee that this large-scale application of an MCIS can further improve the quality, efficiency, and equity of MCH services in Inner Mongolia and provide valuable experience to medical administration departments, practitioners, and medical informatic constructors worldwide.

## Abbreviations

B/S: browser/server
CC and BC: cervical cancer and breast cancer
HIS: hospital information system
IoT: internet of things
LIS: laboratory information system
MCH: maternal and child health
MCHH: Maternal and Child Health Handbook
MCIS: maternal and child information system
MMR: maternal mortality rate
NHC: National Health Commission
NMR: neonatal mortality rate
RMCIS: regional maternal and child information system
UMR: under-5 mortality rate
WHO: World Health Organization

## Data Availability

The data sets including patients’ individual information are not publicly available due to privacy protection and data security regulations. With the consent of the local health commission, the technology company in charge of system development and maintenance generated summary data according to the analysis requirements for this study, which have all been included in this paper.
